# A critical ethnographic study of discriminatory social practice during clinical practice in emergency medical care

**DOI:** 10.1186/s12913-021-06829-y

**Published:** 2021-08-10

**Authors:** Tshepo Nelson Maake, Bernadette Theresa Millar, Lloyd Denzil Christopher, Navindhra Naidoo

**Affiliations:** 1 Faculty of Health and Wellness Sciences, Department of Emergency Medical Science, Cape Peninsula University of Technology, Cape Town, South Africa; 2 Emergency Medical Rescue College, Mbabane, Kingdom of Eswatini; 3 School of Health Sciences: Paramedicine, Western Sydney University, Sydney, Australia

**Keywords:** Racism, Discrimination, Black students, Work-integrated learning, Micro-aggression, Social Inclusion, Emergency Care, Paramedicine Education

## Abstract

**Background:**

Post-apartheid, South Africa adopted an inclusive education system that was intended to be free of unfair discrimination. This qualitative study examines the experiences and perceptions of racial discrimination between Emergency Medical Care (EMC) students, clinical mentors, and patients within an Emergency Medical Service (EMS) during clinical practice. Understanding the nature of such discrimination is critical for redress.

**Methods:**

Within the conceptual framework of Critical Race Theory, critical ethnographic methodology explored how discriminatory social practice manifests during clinical practice. Semi-structured interviews enabled thematic analysis. We purposively sampled 13 undergraduate EMC students and 5 Emergency Care (EC) providers.

**Results:**

EMC student participants reported experiences of racial and gender discrimination during work-integrated learning (WIL) as they were treated differently on the basis of race and gender. Language was used as an intentional barrier to isolate students from the patients during WIL because EC providers would intentionally speak in a language not understood by the student and failed to translate vital medical information about the case. This conduct prevented some students from engaging in clinical decision-making.

**Conclusions:**

Unfair discrimination within the pre-hospital setting have an impact on the learning opportunities of EMC students. Such practice violates basic human rights and has the potential to negatively affect the clinical management of patients, thus it has the potential to violate patient’s rights. This study confirms the existence of discriminatory practices during WIL which is usually unreported. The lack of a structured approach to redress the discrimination causes a lack of inclusivity and unequal access to clinical education in a public clinical platform.

**Supplementary Information:**

The online version contains supplementary material available at 10.1186/s12913-021-06829-y.

## Background

The exploration of racial discrimination appears to have little presence (or voice) in the EMC discourse. However, within the South African healthcare sector some studies have documented discrimination experienced by medical students during their hospital clinical attachments. In a study conducted by Thackwell et al. [1], black trainee participants reported experiencing racism at some point during their training and indicated that it was not overt racism, but covert racism which collectively accumulated into feeling unwanted within the medical working environment.

However, as an undergraduate National Diploma in Emergency Medical Care (NDip EMC) student, I [TNM] observed and (rather painfully) experienced discrimination with fellow EMC students during time in clinical training practice within the pre-hospital setting in Johannesburg and Cape Town. Later, in my Bachelor of Technology in Emergency Medical Care (BTech EMC) undergraduate research project, racial discrimination was one of the themes that emerged as participants shared their experiences in clinical practice. Through the theoretical framework of Critical Race Theory, therefore, this study documents the existence and extent of racial (and other) discrimination within the Emergency Medical Service (EMS) in South Africa.

When addressing overt or covert racism, it is useful to consider some of the definitions provided in the academic literature. Stevens [2] defines racism as an unsupported belief that among humans there are biological hierarchies in the form of different races and attempts to justify the political, economic and social exploitation of certain social groups by others. Phiri and Matambo [3] argue for the use of ‘Fanonian analysis’ in that the credibility given to the conception of race has been overrated, as the argument associated with the conception of race or racism gave it an ontological significance, thus making ‘race’ a standard factor to decide who is human and who is not.

Critical Race Theory (CRT) [4] views race as a political and social construct that may be manipulated by society when it is convenient. In addition, CRT considers racism to be a social norm that is a common everyday experience for a black person [5]. CRT also states that racism is not the same as discrimination, hatred or racial prejudice because racism involves a series of systems embedded through institutional policies and practices in society that ensure that one group has the power to discriminate [5].

“Everyday racism is racism, but not all racism is everyday racism. From everyday racism there is no relief” [6]. This statement is helpful in unpacking covert racism which happens when black people (regardless of class or culture) are subjected to vicarious racism and the indignities of micro-aggressions that contribute to racism-related stress [7]. Greene and Blitz [8] suggest that white people’s inability to relate to micro-aggressions experienced by people of colour renders them unable to be empathetic to their friends and colleagues who are people of colour. Solórzano, Ceja and Yosso [9] found that this overt racial microaggression and stereotyping resulted in African American students underperforming academically to the point of eventually having to drop a class, leave the institution for somewhere else or change their major.

In 2019, the South African Human Rights Commission (SAHRC) [10] found that in the past 20 years the public universities in South Africa have failed to sufficiently transform, and discrimination remains prevalent on the basis of gender, race, disability and socio-economic class. There exists, therefore, a disjuncture between institutional policies against discrimination and real-life experiences of students and staff.

To understand the pervasiveness of racial and other forms of discrimination, we have used the lens of intersectionality. Systemic injustices and social inequalities occur on multifaceted levels, intersectionality contends that the traditional notions of oppression such as racism, sexism and homophobia are not independent. Rather these interrelate and generate a system of oppression that resonates the “intersection” of multiple forms of discrimination” [11]. Social inequality and systemic injustices occur on multi-faceted levels, and we can comprehend that traditional notions of oppression such as racism and discrimination on the basis of gender are not independent of each other but may occur simultaneously interconnected.

In addition to intersectionality, there is privilege that aids and abets racism. McIntosh [12] documents five basic aspects that describe privilege: (i) it is a unique advantage that is not normal; (ii) the individual does not earn it by talent or merit, it is granted; (iii) it is an entitlement that comes in relation to a preferred status; (iv) it is exercised to benefit the recipient but to the exclusion of others, and (v) the person possessing a status of privilege is often not aware of it. Mathews [13] focused on white privilege in South Africa, providing an understanding about the persistence of white privilege in the post-apartheid period, i.e. from 1994 to the present. McKaiser [14] argues that even though not all white people are perpetrators of anti-black racism, they all benefited, and they still do. He describes this as ‘unearned privilege’.

Historically during the apartheid era, the country was divided on the basis of race and majority of the black people had limited access to basic education (known as ‘Bantu Education’) and few in this system were afforded the opportunity to access tertiary education [15] compared to their white counterparts. This obviously created an imbalance in employability as most black people were limited to jobs which did not require any set skills that had to be studied at an institution of higher learning, making most unskilled workers. The few skilled workers at that time would be those who had studied at a separate black university for a specific discipline or specialised in a specific skill; for example, a doctor, teacher or nurse [16]. Racial discrimination had an impact on the overall training of black students enrolled into the university to study medicine during the apartheid era. Black medical students were not allowed to treat white patients during their work-integrated learning; and some hospitals also refused black students entry to white patient wards [17].

With the ‘death’ of apartheid and the advent of the free South Africa, the education system underwent a radical change to become inclusive, especially in higher education, where some historically white universities were merged with historically black universities and technikons in an effort to level the playing field. New universities of technology were created through these mergers. A massive process of recurriculation happened alongside the merger process. New subjects and degrees were on offer to all students. However, the student protest movement “Rhodes must fall” (#RMF) and the Movement to Decolonize the University indicated black students’ awareness and acknowledgement of the existence of institutional racism and patriarchy [18]. Thus, discrimination, intersectionality and privilege were still active as ‘counter-revolutionary’ phenomena 25 years after the demise of apartheid, even within institutions of higher learning and affected students in their learning environment and well as their subjects which required work-integrated learning (WIL).

Within the EMC curriculum, work-integrated learning is a very important component which is featured within the subject called Clinical Practice. EMC students are placed with EC providers, such as ambulances, hospital emergency rooms, response vehicles to gain work experience in a real-life work environment. According to the Health Professions Council of South Africa (HPCSA) [19] qualified and registered EC providers are expected to offer EMC to all patients in need without subjecting the patient to any form of racial discrimination. Failure to do so is a violation of the patient’s rights and undermines adequate patient management. All EMC students in South Africa are required to register with the HPCSA and adhere to its protocols.

The aim of this study was to explore through Critical Race Theory, how discriminatory social practice manifests during clinical practice interactions between emergency medical care students and clinical mentors.

## Methods

In this qualitative research a methodological lens of critical ethnography is used with a layer of autoethnography [20]. Critical Race Theory provides the analytical frame to help make meaning from the data. This study focused primarily on racial discrimination within the pre-hospital setting during EMC students’ clinical practice. This context focused on the relationship between EMC student and EC providers, EC providers and patient, as well as EMC Student and patient. The aim of the study was to explore, through CRT, how discriminatory social practice manifests during clinical practice interactions between EC students and clinical mentors and how it was experienced or perceived by both the EC provider and the student.

A purposive sampling strategy was used for this study, provided inclusion criteria were met. Data was collected from 5 qualified EC providers currently registered with HPCSA and practising within the South African EMS. Data was also collected from 13 final-year EMC students. These were considered appropriate participants as they were actively practising within the pre-hospital setting and engaged in clinical practice with more than three years’ clinical experience during work-integrated learning [21].

A face-to-face, in-depth semi-structured interview approach was used, thus allowing participants freedom and willingness to share experiences openly [22]. Interviews were held at a private research venue in the University. Participants were asked to come in for the interview at a date and time convenient to them so as not to affect their learning schedule. For Emergency Medical Practitioners currently practising, interviews were also held at their workstations unless preferred otherwise by the practitioner. In that situation, another date, time and place were set for the interview that did not compromise service delivery if the participants were on duty. Interviews, with the aid of an interview guide, were recorded for later transcription; interviews were between 20 and 45 min.

Through the use of CRT to break down the data, concrete descriptions were derived to classify into patterns and themes which addressed CRT categories. The use of concept mind-mapping represented ideas linked around a central theme [23]. Categorising and Coding depended on what was meaningful to the research topic and what would assist in responding to the research question [21].

The Critical Race Theory was utilized as analysis tool that allowed the author to consider socioecological experiences and perspective which focused on racial attitudes and beliefs that permeates within institutions (Tertiary Institutions and Emergency Medical Service base stations), healthcare in the prehospital sector, community and policies/law. CRT therefore provided an opportunity to view the healthcare community and training institutions as part of an organ that exist within a society that bears the impact of a racially discrimination and injustice. The construct in CRT also guided the study through providing the author with the opportunity to recognize complex intersections and relationships that exist within race, gender and language amongst students during their clinical attachment.

The Research Ethics Committee provided institutional ethical clearance for the study to be conducted (Ref: HW-REC 2016/H28) on the basis of criticality of apartheid classifications and potential for redress.

## Results

The results will be highlighted with direct quotes from participants’ in this study.

The three main themes and seven subthemes derived through the open coding process from the transcriptions are listed in Table 1 and discussed below.

**Table 1:** Themes and subthemes

MAIN THEMES	SUB-THEMES
1. Uncovering an EC Provider’s Gender Constraints	1a. Sexual harassment. 1b. Gender inequality and patriarchy.
2. Being my ‘Race’ within the Institution	2a. A discourse between racism and preference. 2b. Perception of race within the EMS industry. 2c. Conceptualization of racism and privilege in EMS.
3. Language discordance in linguistically diverse clinical settings	3a. EMS and language dynamics 3b. Language barrier during work-integrated learning and patient care.

### Theme 1: uncovering an EC provider’s gender constraints

Historically, the EMC profession has been dominated by male EC providers. Critical Theory assumptions are helpful here as they posit that knowledge is historically constructed. The introduction of female practitioners into the profession is a step in the direction of gender equality in the workspace. The pre-hospital EC setting is an uncontrolled environment which further exposes female EC providers to various challenges, such as sexual harassment.

### Subtheme 1a: sexual harassment

*With that free hand he tried to grope me, which was very inappropriate, and I was like, what are you doing? … he was just speaking to me in a…very sexual behaviour. (EMC Student)*

The white female student participant above experienced sexual harassment perpetrated by the patient, where the harasser used grossly sexually offensive and threatening language towards the student, thus creating a hostile environment.

*He said to me that both him and the patient were going to rape me… I pictured them physically raping me and killing my partner… so my imagination was there. Even though it didn’t happen, it went there and the fact that his hands were in my panty… (EC provider).*

The female Person of Colour (Coloured) participant above had experienced sexual harassment and even threats of gang-rape during her clinical practice. These threats were made by the person accompanying the patient. Conventionally, one person is allowed to escort the patient to the hospital and they usually accompany the patient alone in the back of the ambulance where one EC provider is on duty. The participant felt as if she *had* been raped by the patient escort because he verbalised malicious intent and then demonstrated his power to do so.

### Subtheme 1b: gender inequality and patriarchy

Gender inequality and patriarchy emerged in relation to risk. During clinical practice in the pre-hospital setting, participants experienced gender inequality and manifestations of patriarchy during their activities in the provision of care.

*There were African practitioners working with me and they said to the control centre we have a white female student with us, we are refusing to enter the zone, the area, (EMC student).*

A white female student participant was working with a non-white crew when she overheard them informing the control centre that they would not be able to enter the zone because they have a white female student. The participant described the area as being hostile, meaning that there was some concern for her safety. In the participant’s view, however, it is unclear if the decision taken by the non-white practitioner was based on her race, gender or because she is a student.

### Theme 2: being my ‘race’ within the Institution

As mentioned in the background, despite political and social transformation, racism appears to remain within South African society. Racial discrimination or racism was either experienced or witnessed by participants and it was apparent to the author that the feeling of being discriminated against on the basis of race was experienced and perceived by both so-called white and non-white EMC students and EC providers.

### Subtheme 2a: a discourse between racism and preference

When the focus was placed on whether the patient’s preference was to be treated by a specific practitioner, this was motivated by racism.

*Black people associate with black people and white people associate with white people and so forth. If I had to compare all those shifts, I feel that black people would treat me nicer compared to your coloured and white people…. (EMC student).*

A student black male participant indicated that there is a subconscious grouping within the student classroom group that divides them into multiple clusters which are predetermined by race or class, even though the class is treated as one homogenous group of individuals by the lecturer. However, within the classroom grouping itself, students do not see themselves as ‘one’. He also indicated a feeling of comfort associated with working with a crew of the same race or ethnic group and who share similar background.

*When I deal with people from my own colour or race (coloured) and black people…I do feel that I don’t have to explain myself… (EC provider).*

As a coloured EC provider, the participant felt comfortable working with practitioners of the same race. There is a sense of belonging when with coloured EC providers compared to when he is with EC white providers.

### Subtheme 2b: perception of race within the EMS industry

*There is just too much discrimination within the EMS. I believe that black people have not been fully accepted in this industry. Most white people still believe that it is their industry and they are the only ones who deserves to be Advanced Life Support practitioner. (EMC Student).*

This black male student feels alienated and unwanted by white people in the EMS. He indicates that white people believe that they should be the only ones who should progress within the EMS industry. What is the historical significance of such an observation? This can be explained as professional entitlement on the basis of race. South Africa, under apartheid, used the mechanism of job reservation to promote white privilege in professions and management. This historical practice has resulted in the need, post-apartheid, for employment equity legislation that outlaws job reservation and promotes affirmative action. Notwithstanding the intention for affirmative action to widen access to opportunities, its backlash has been to stigmatise black appointments as a procedural requirement rather than one of merit. In EMS, such stigma is indefensible as all graduates must pass HPCSA accredited programmes and hold professional registration.

### Subtheme 2c: conceptualization of racism and privilege in EMS

In attempting to document the existence of racial discrimination within the EMS, a correlation arose between racism and privilege in the EMS.

*A lot of people still assume that because you are white, you are privileged. It is a fair assumption because most of the white people in the country are privileged (EMC student).*

This white male student continues to draw a correlation between white people and privilege. He accepts the assumption made by majority of black and coloured people who view white people as being privileged and agrees that it is a fair assumption.

### Theme 3: language discordance in linguistically diverse clinical setting

South Africa is a multicultural, multi-lingual, and diverse country with eleven official languages, all equal before the law. To examine whether the concept of language has an impact on EMC students during their work-integrated learning, this study considered language a fundamental basis for the transfer of knowledge and skill between qualified EC providers and students.

### Subtheme 3a: EMS and language dynamics

The pre-hospital environment exposes the student and Emergency Care providers to different language dynamics. These dynamics range from having to communicate with a patient using any of the 11 official languages in South Africa to the crews deciding on which language to use in their workplace or in their respective emergency vehicle. The dynamics are made more complex by the periodic introduction of a student, who mother-tongue may be different, into the emergency vehicle for the purpose of work-integrated learning.

*I told them that I do not understand Afrikaans, and they said no this is how we talk in this ambulance and there is no way that we are going to speak in English….they were giving me orders in Afrikaans and I was just thinking maybe this is what they want me to do and what if I do the wrong thing. (EMC student).*

The student above, as a black non-Afrikaans-speaking female, felt disconnected by the crew (EC providers) because of the language barrier when the crew made it clear that they would not change their language to accommodate her. During clinical practice she was also given instructions in Afrikaans and she constantly feared making mistakes that could harm patients.

### Subtheme 3b: language barrier during work-integrated learning and patient care

The difference in the languages spoken by the student and the EC provider may also pose a barrier between the two. Hence, socio-cultural competence is needed.

*The Xhosa speaking students that were there, uhm…were allowed to perform skills because, they spoke in Xhosa and they would ask questions and if I spoke in English they wouldn’t acknowledge me (EMC student).*

As a white female student working with a crew that spoke Xhosa, the student above indicated that she was denied the opportunity to perform a skill during clinical practice. Instead the EC providers availed that opportunity to Xhosa-speaking students who were also on shift. The difference in spoken languages made her believe that if she could speak isiXhosa the EC provider may have afforded her the opportunity to perform the skill but because of the language barrier she lost a learning opportunity.

## Discussion

Overall, participants did experience some form of discrimination during their clinical practice. Key themes were interpreted through a Critical Race Theory (CRT) lens to understand how discrimination manifests during work-place learning in the pre-hospital setting and also how students and Emergency Care providers navigate this tense atmosphere of discrimination.

### Uncovering a paramedic’s gender constraints

A conceptual difference exists between the terms ‘gender’ and ‘sex’. Gender refers to the social or cultural constructs associated with being male or female whilst sex refers to the physiological, physical differentiations, the reproductive system, height and masculinity/femininity of an individual [24].

#### Sexual harassment

Sexual harassment can occur in a patriarchal system model where the harassment can be explained in a societal context. This model explains that sexual harassment occurs due to male dominance over women [25]. The case of the patient who sexually harassed and assaulted the caregivers has reference. The patient’s actions were experienced as grossly violating and violently intrusive towards the practitioner. The patient, therefore, violated the EC provider’s rights according to South African legislation. Moffet [26] explains the occurrence of sexual violence is due to South Africans experiencing a toxic masculinity crisis where they use sexual violence to keep women in subordinate positions. Until the 18th century, little or no attention was paid to social inequality [27]. More recently, the implications for gender inequality in the workplace are of particular interest.

#### Gender inequality and patriarchy

There is a need to balance employment rights in patient care. The role players in this instance viewed it ethically justifiable that EMS personnel were refused entry into an area if it compromised their safety. It remains unclear if EC providers would have proceeded to go into a hostile area if they were working with either with a black female student, or a black male student, or a white male student or with no student, but at face value it appears that judgment was made on the basis of safety for the student rather than discrimination or bias. This situation may also demonstrate the presence of intersectionality [11] in decision-making within the EMS.

The safety of the crew remains a priority when entering any emergency situation. Recent multiple attacks in the Western Cape EMS made Paramedics more vigilant when attending to incidents in the pre-hospital setting. A newsletter from the Emergency Care Professional Board indicated attacks against EMS personnel are on the rise with 46 attacks reported between January and 18 September 2020 in the Western Cape Province alone, in South Africa [28].

### Being my ‘race’ within the institution

#### A discourse between racism and preference

When considering the discourse between racism and preference, the following were noted. Patients may prefer healthcare provider-patient racial concordance; where the patient’s refusal to be treated by a healthcare provider may be racially motivated due to the belief that medical treatment provided by a black healthcare provider is of poor quality. According to the Health Professions Council of South Africa [19], the patient has the right of choice to health services. This gives the patient freedom to choose a specific health care provider to offer them a service or treatment at a facility of their choice. The right is only granted provided it does not contradict the ethical standards applicable to the health care provider or facility. What often remains unclear is whether the patient’s refusal of care is based on the assigned healthcare provider’s racial identity or some other reason. This practice poses a dilemma between medicine and ethics since satisfying the patient’s preference may be viewed as supporting racial discrimination and would be against the Bill of Rights in the South African constitution.

Prior 1994 South African healthcare professional were conflicted with human rights abuse, this was also demonstrated through the death of Steve Biko and how healthcare providers who managed his case conducted themselves. Majority of the black South African’s right to healthcare has systematically undermined due discriminatory practices which has been happening for years [29].

There seems to be an expectation of more room to learn and share knowledge between student and mentor and patient and healthcare provider when the two parties involved are from the same race. A culture of racial disparity exists amongst students in the same class as racial grouping. There is also a perceived level of comfort that a student has when he works under supervision of someone of his own race. The racial disparity is evident between practitioner and patient.

#### Perception of race within the EMS industry

Participants mentioned that white people seem to believe that the EMS industry ‘belongs’ to them because they are in the majority as a result of past unfair advantage in access to education and training facilities. The shortage of black EC providers in the field trained at an Advanced Life Support level seems to affirm this white majority. Does this dominance equate to a misinformed sense of ownership (and cultural hegemony) that excludes the marginalised from the environment?

Karlberg [30] deconstructs the dominance of power and clarifies elements of an alternative discourse of power. The study exposes the distinctions of ‘power over’ which are shown in issues of control, social conflict and coercion. By applying Critical Race Theory, a correlation between majority, dominance and power was constructed to see if there is a relationship between each of them. This could assist in understanding why a certain group within a population dominated by one race would feel marginalized.

#### Conceptualization of racism and privilege in EMS

To conceptualise the idea of racism and privilege within the Emergency Medical Service it is essential to demonstrate a clear understanding of both racism and privilege respectively. This allows for an interpretation of this phenomenon collectively from a CRT point of view. Within a racist-orientated society, individuals will express some form of racialization. The process of racialization is vital in identifying the difference between ‘self’ and ‘other’. A Coloured female is negatively perceived as ‘other’ as she was identified as black (making her feel inferior because of race). This indicates that despite constitutional changes, South Africans remain divided on the basis of race with a constant but covert undertone of racial discrimination. With a CRT convergence lens, we uncover participants’ experience through Intersectionality.

In the deployment of Critical Race Theory, a broader appreciation of privilege is required to understand if the participant’s statement that white people are privileged is a fair assumption to make. In the context of EMS it can be seen where patients are more open to talk to the white student and also assume the white student is the doctor. In this way, the patient has unwittingly given the student a higher status in the presence of two other qualified black EC providers supervising the student and it would appear in this case to be a matter of respect for white privilege, which as Mcintosh [12] indicates, is ‘unearned power’ which is not offered on the basis of merit or intelligence but because of race.

### Language discordance in linguistically diverse clinical settings

South African institutions of higher learning are largely made up of diverse student groups who speak various languages. According to Keeton et al. [31], clinical practice is the fundamental element of Health Science programmes as it ensures students acquire knowledge and skills transfer in linguistically diverse clinical settings. Practitioners have a dual obligation during clinical practice; as clinician and caregiver to the patient and as mentors they have to afford students learning opportunities during their clinical practice attachments.

#### EMS and language dynamics

What participants have highlighted is the correlation between language barrier and productivity of the shift. Participants predicted an unproductive shift on their arrival at the base station based on language barriers between them (the student) and the EC provider.

This intentional exclusion of an EMC student by an EC provider and subsequent lack of consideration made the student feel discriminated against on the basis of language. This behaviour would be something beyond the student’s control and does not form part of their intended training; however, the only way to overcome that imbalance would be for the student to speak Afrikaans. This then becomes an unfair discrimination because it disadvantages some; only those students privileged enough to have Afrikaans as their first or second language could have a productive shift and engage academically with the crew to learn as much as possible on the shift.

#### Language barrier during work-integrated learning and patient care

Participants reported occasions whereby the Afrikaans EC providers would speak to each other in Afrikaans the entire shift, and if they happen to be dispatched to an Afrikaans-speaking patient, they would continue speaking to the patient in Afrikaans and would not bother explaining/translating the patient’s condition or symptoms to the student. This conduct, therefore, totally excluded the students from participating in patient care.

Participants further indicated that while mentors did not feel compelled to translate information to accommodate the student, by contrast EC providers were happy to offer an opportunity to a student who spoke the same language as them. Participants experienced this approach discriminatory because only some students with a particular language characteristic could benefit from the clinical attachment shift, to the exclusion of others. Such practice contributes to the ‘othering’ of students, a basis for social exclusion. Using a language that the patient understands contributes positively to the patient’s diagnosis and clinical interventions. In the context of experiential learning, perhaps the most appropriate method of approach would be for the EC provider to accommodate the student by sharing with them the clinical presentation of the patient so that they also could be in a position to learn from that particular case and suggest a treatment plan, as expected by their curriculum.

## Conclusions

In an attempt to contribute to change and redress in a post-apartheid, racially and culturally diverse society whose Constitution [32] condemns unfair discrimination, this study considered Emergency Medical Care (EMC) culture within one province of South Africa through the lens of prestige, privilege, power and authority. The aim was to document discriminatory social practices during EMC education and practice specifically in the context of EMC students engaged in work-integrated learning during their clinical practice. Although focus was placed on racial discrimination, other forms of discrimination emerged during the data analysis, resonating with the work of Bonilla-Silva et al. [33]. In addition, this study, framed by Critical Race Theory, demonstrates the impact of discriminatory practices on EMC students. This leads to an understanding of how racial discrimination is experienced or perceived by victims. This study, therefore, contributes to the racism discourse from a transformative stance.

This study provides tentative evidence to support the existence of discriminatory practices during clinical practice in work-integrated learning as experienced by students and EC providers. The impact of racial discrimination is explored (as experienced by students as victims or perpetrators and EC providers as victims or perpetrators) through Critical Race Theory. Racism experienced by participants is described as covert racism which makes it difficult to address in the dynamic EMS workplace.

Gender and language are also reported as barriers that affected student’s learning opportunities and were reported to discriminate against EMC students during work-integrated learning thereby impacting on the student’s clinical practice and exposure. Collectively these experiences had an impact on the viability of students being able to extract knowledge from the mentor or the mentor creating an educative environment for the student. It may be true to say that the current *modus operandi* of some mentors represents ‘tormentor-ship’ rather than mentorship.

The distinctive features of South African history that contribute to the current problems include racial, gender and language discrimination. These are challenges deeply rooted within South African society and also embedded in the EMS industry, starting from when participants are EMC students until they are qualified and working within the EMS. This behaviour has had a negative impact on EMC student’s experiential learning.

Cognitive dissonance is created by unfair discrimination by EMC students, Patients and EC providers. This paper documents this dissonance to advance the professional and public interest in social justice. Therefore, a comprehensive mentorship programme should be created to consider objective and subjective criteria for mentors and student selection, to achieve expectations validated by educational theory and formal assessment, and to perform and effectively measure the mentoring relationship/programme. These deliberate measures are necessary to respond to the counter-productive experience of discriminatory social practice during clinical practice and the subsequent risk of post-apartheid styled job reservation.

## Supplementary Information

**Additional file 1.** Interview guide

## Data Availability

Anonymised data is available from the corresponding author upon request.

## Abbreviations

BEMC: Bachelor of Emergency Medical Care
BTech EMC: Bachelor of Technology in Emergency Medical Care
CRT: Critical Race Theory
ECP: Emergency Care Practitioner
EMC: Emergency Medical Care
WIL: Work-Integrated Learning
EMS: Emergency Medical Service
HPCSA: Health Professions Council of South Africa
