# Supplementary material for: A critical ethnographic study of discriminatory social practice during clinical practice in emergency medical care
Source: BMC Health Serv Res. 2021 Aug 10;21:787. doi: 10.1186/s12913-021-06829-y (PMC8353834; doi:10.1186/s12913-021-06829-y)
Supplement: Supplementary file 1 — Additional file 1. Interview guide [file 12913_2021_6829_MOESM1_ESM.pdf]

INTERVIEW GUIDE

Time of interview : ______________________

Date : ______________________

Place : ______________________

Interviewer : ______________________

Interviewee : ______________________

Welcome and introduction - 2 minutes

Opening question and discussion - 15 - 45 minutes (unlimited if necessary)

Interview Question for students - **“share your discriminatory experiences or perceptions during work-integrated learning/clinical practice as an Emergency Medical Care student?”**

Interview question for EC providers -**“share your discriminatory experience or perceptions during clinical practice as an Emergency Care provider?**,

Closure - 2 minutes

Notes : (Hand written during the interview)

……………………………………………………………………………………

……………………………………………………………………………………

……………………………………………………………………………………

……………………………………………………………………………………

Follow up **prompts** that may be used "whenever necessary":

- "Why do you think this is the case?"
- "What causes this?"
- How did you deal with it?
- "Why do you think this is the case?"

"Can you perhaps elaborate more on that
